# Staying grounded in turbulent times: the power of mindfulness for maintaining mental well-being during COVID-19

**DOI:** 10.47626/2237-6089-2023-0699

**Published:** 2025-01-07

**Authors:** Kyara Rodrigues de Aguiar, Marília Silva de Souza

**Affiliations:** 1 Centro de Pesquisa Experimental Centro de Pesquisa Clínica Hospital de Clínicas de Porto Alegre Porto Alegre RS Brazil Laboratório de Psiquiatria Molecular, Centro de Pesquisa Experimental (CPE), and Centro de Pesquisa Clínica (CPC), Hospital de Clínicas de Porto Alegre (HCPA), Porto Alegre, RS, Brazil.; 2 Curso de Pós-Graduação em Psiquiatria e Ciências do Comportamento Departamento de Psiquiatria, Faculdade de Medicina Universidade Federal do Rio Grande do Sul Porto Alegre RS Brazil Curso de Pós-Graduação em Psiquiatria e Ciências do Comportamento, Departamento de Psiquiatria, Faculdade de Medicina, Universidade Federal do Rio Grande do Sul, Porto Alegre, RS, Brazil.; 3 Faculdade Anhanguera de Pelotas Pelotas RS Brazil Faculdade Anhanguera de Pelotas, Pelotas, RS, Brazil.

During the 1st year of the pandemic, there was a 25% increase in the prevalence of depression and anxiety worldwide.^1^ This rise was accompanied by an uptick in suicide risk, highlighting the impact of the coronavirus disease 2019 (COVID-19) pandemic on mental health.^2,3^ This rise was accompanied by an uptick in suicide risk, highlighting the impact of the COVID-19 pandemic on mental health.

Even before the pandemic, mental health had been a pressing issue that warranted greater attention and investment. The disparities between the population’s mental health needs and available services have not been adequately addressed, perpetuating a chronic crisis that has been exacerbated by the pandemic.^4^

As the pandemic progressed, we witnessed a series of grave challenges, including higher rates of infection and mortality, economical burning, and ambiguous messages from leaders around the world.^5^ These challenges contributed to increased anxieties and concerns about the pandemic’s potential effects on mental health. Furthermore, public health strategies were politicized in some countries, leading to greater uncertainty, morbidity, and mortality.^6^

This critical time of difficult assimilation and transition was particularly difficult for certain populations who were even more vulnerable to the consequences of COVID-19. These populations are the ones that were already at a higher risk for mental illness due to several risk factors that have been documented in the literature for serious mental health outcomes, including suicide.^7^ Specifically, these groups include individuals from marginalized racial/ethnic and sexual communities, those facing economic disadvantage, and those who have limited access to healthcare.^7^ The pre-existing social, economic, educational, and health disparities were accentuated by the pandemic. Yet, in the pandemic setting, the high-risk group also included frontline healthcare workers and essential workers, who in addition to being at high risk of contracting the virus, were also susceptible to mental illness.^8,9^

In summary, the pandemic highlighted the pressing need to address the chronic crisis in mental health that has plagued society for decades. While COVID-19 has exacerbated the problem, the pandemic has only brought existing mental health issues to the fore, magnifying disparities that have not been sufficiently addressed. From a broader perspective, this scenario presented a new and urgent opportunity to shift political and federal resources and investments towards mental health issues that could no longer be overlooked.^10^ Numerous studies have been conducted to identify the impacts of the pandemic on mental health and the human brain.^11^ To grasp the importance of suitable interventions that can enhance mental health care, this editorial will center its attention on mindfulness-based interventions (MBIs), which has gained prominence as an effective strategy for promoting mental health.^12-14^

Mindfulness is usually defined as the awareness that arises through paying attention to the present moment without judgment.^15^ The mind is trained to stay in the present moment, open, calm, and alert, contemplating the moment. Mindfulness is basically a particular way of paying attention. It is a way of looking deeply inside yourself, with a self-inquiry and understanding yourself.^15^

The term “mindfulness” is the translation of *sati*, which in Pali (language used in the Buddhist sacred writings) means remembrance or remembering.^16^ Mindfulness is one of the focal points of the Buddhist teachings.^17^ In this perspective, it is an active and investigative practice or process that involves above all cognitive, attitudinal, affective, and even social and ethical dimensions.^18^ Conscious attention and perception are actively cultivated and can be developed through the systematic habit of meditation.^19^

Mindfulness comprises two key components: cognitive processes focused on actively managing one’s immediate experience, and emotional processes involving the cultivation of a curious, open, and accepting stance towards present-moment sensations.^19^ The practice of mindfulness meditation and other forms of meditation is gaining popularity, with an increasing number of individuals incorporating them into their lives to achieve diverse outcomes.^20,21^ Mindfulness practices have been imparted in non-religious settings, such as educational institutions, hospitals, and clinical settings.^22,23^ Additionally, they have found their place in the academic and scientific environment.^24-26^

Joh Kabat-Zinn is considered one of the pioneers in the expansion and use of mindfulness in health services. He started, in 1979, a program that today is called Mindfulness-Based Stress Reduction (MBSR).^27^ The MBSR was developed in order to help individuals learn to cope with pain, stress, and illness. It is a patient-centered educational program that includes intensive mindfulness training to teach them how to take better care of themselves and live healthier, more adaptive lives.^28^

Since the inception of this protocol, over 740 MBSR programs have been developed based on the original model.^29^ The efficacy of MBIs has been extensively researched and shown to have positive effects on both mental and physical health in clinical and non-clinical populations.^30-32^ It has demonstrated its ability to reduce emotional reactivity by cultivating a focused awareness of thoughts and emotions.^33^ Furthermore, mindfulness is recognized as a strategy to enhance emotional well-being and cope with various challenges such as stress, depression, anxiety, pain, and substance abuse.^34,35^

Mindfulness can be embraced through formal meditation practices as well as informal awareness of each moment throughout the day.^36^ Most MBIs involve a structured program spanning 2 to 3 months, with weekly sessions (typically eight-12 sessions) and encouraged regular home practice. However, there is no consensus in the literature regarding the minimum duration required or the optimal dose of mindfulness practice to yield benefits. While it is hypothesized that longer and more regular practice may lead to greater benefits, evidence suggests that the rate of improvement tends to plateau with increased sessions.^37^ Instead, the amount of formal practice conducted between sessions, rather than the duration of individual sessions or the overall program length, has been found to be a more accurate predictor of outcomes and benefits.^38,39^ Continued mindfulness practice is known to foster higher levels of self-compassion, cultivate empathy and compassion towards others, regulate stress, and enhance effective coping with stress.^40^

In a pre-pandemic context, MBI proved to be a viable and effective treatment for reducing the risk of suicide.^41^ Specifically Mindfulness-Based Cognitive Therapy (MBCT) for patients with mood disorder, there are several high-quality studies that support its impact in reducing the risk of suicide.^41^ Given the current global situation and the growing concern around maintaining good mental health and reducing stress and anxiety during quarantine periods, a number of studies have started to explore the effectiveness of interventions that have previously shown success in managing various psychiatric conditions in the context of COVID-19. A systematic review has compiled randomized clinical trials (RCT) conducted during the pandemic, which investigated the effectiveness of online interventions in addressing anxiety, depression, stress, or subjective well-being in humans.^42^ The review identified that mindfulness-based practices were effective in reducing anxiety, depression, and stress while also increasing subjective well-being when compared to both active control groups (those receiving comparable treatments) and inactive control groups (those on a waitlist).^42^ Also, for subjective well-being, the study found a tendency for MBI to show larger effect sizes compared to cognitive-behavioral therapy (CBT) (b = 0.16 [95% confidence interval {95%CI} 0.02 to -0.34], p = 0.086).^42^

Two additional systematic reviews focused on studies conducted during the pandemic period, covering from January 2020 to May 31, 2021, while another review considered studies from January 2020 to March 2022. These reviews specifically investigated the impact of MBIs in reducing symptoms of depression, anxiety, and stress during the pandemic.^43,44^

The results of both reviews showed a significant decrease in these symptoms. Additionally, one of the studies conducted a meta-analysis, which revealed small to moderate reductions in depression, anxiety, and stress levels following the intervention.^43^ The researchers also observed notable effects of online MBIs on depression and anxiety during the follow-up period, indicating that such interventions may have medium-term benefits for enhancing mental health.^43^ These positive outcomes may be attributed to the association between mindfulness and acceptance skills, which may promote greater mental health resilience.

In a similar direction, another systematic review was developed to investigate whether mindfulness, within the context of public mental health, can serve as a protective factor against depression and anxiety disorders related to the COVID-19 pandemic.^45^ The results demonstrated in this study suggest that there is a negative correlation between mindfulness and both anxiety and depression. The negative correlation values between mindfulness and anxiety and depression were -0.330 and -0.353, respectively, which indicates that higher levels of mindfulness are associated with lower levels of anxiety and depression.^45^ When the study is focused solely on the effects of mindfulness practice on anxiety, the relationship is stronger (-0.445; 95%CI -0.537 to -0.343; k = 4). On the other hand, when moderator variables (region and the effective form of the mindfulness) indirectly influence anxiety levels, the effect size is more modest (-0.265; 95%CI -0.402 to -0.116; k = 8).

Additionally, the meta-analysis showed that the correlation between mindfulness and anxiety varied depending on the study region. The correlation was stronger in Europe (-0.457; 95%CI -0.512 to -0.398; k = 8) than in North America (-0.070; 95%CI -0.136 to -0.002; k = 4), indicating that cultural, social, or other regional factors may play a role in this relationship.^45^ It is important to note that the studies included in the analysis were mainly conducted in regions with developed economies. The authors draw attention to the shortage of research in low-income countries, such as Africa, where access to vaccines was limited and the impact of the COVID-19 pandemic on people’s mental health could be more significant. Importantly, the type of sample (clinical, community, and college samples) did not significantly affect the association between mindfulness and depression or anxiety, according to the findings.

Overall, available literature data suggest that skills taught in MBI may be useful in managing various forms of distress, including the psychological impact of the pandemic. However, it is worth noting that cultural or regional differences may also play a role in the effectiveness of MBI. Future research should prioritize examining the role of mindfulness and mental health particularly in developing nations. Also, the effectiveness of MBI in marginalized racial/ethnic and sexual groups, as well as economically disadvantaged populations, should also be explored. These populations may be experiencing higher levels of stress due to exposure to various stressors, such as economic instability and limited access to healthcare. Therefore, to ensure the effectiveness of MBI, it is crucial to consider the various stressors and challenges faced by these populations. The highlighted findings can be applied in a post-pandemic context by utilizing MBI skills to address psychological distress. Recognizing cultural differences remains crucial, and this data encourages further research on mental health in developing nations and among marginalized groups experiencing chronic stressors.
